# Antioxidant defence and oxidative stress markers in cats with asymptomatic and symptomatic hypertrophic cardiomyopathy: a pilot study

**DOI:** 10.1186/s12917-020-2256-3

**Published:** 2020-01-30

**Authors:** Marcin Michałek, Aleksandra Tabiś, Urszula Pasławska, Agnieszka Noszczyk-Nowak

**Affiliations:** 1 Department of Internal Medicine and Clinic of Diseases of Horses, Dogs and Cats, Faculty of Veterinary Medicine, Wrocław University of Environmental and Life Sciences, Grunwaldzki sq. 47, Wrocław, 50-366 Poland; 2 Department of Food Hygiene and Consumer Health, Faculty of Veterinary Medicine, Wrocław University of Environmental and Life Sciences, C.K. Norwida 31, Wrocław, 50-375 Poland

**Keywords:** Cardiology, Cats, Hypertrophic cardiomyopathy, Oxidative stress, Antioxidative enzymes

## Abstract

**Background:**

Hypertrophic cardiomyopathy is the most common cardiovascular cause of death in cats. Although the majority of cats remain asymptomatic, some may develop signs of chronic heart failure due to diastolic failure, arterial thromboembolism (ATE) or sudden cardiac death. Therefore, it is crucial to identify individuals that are in high risk of developing cardiac complications before the onset of life-threatening signs. Oxidative stress is the imbalance between the production and neutralisation of reactive oxygen species. Uncontrolled reactive oxygen species overproduction leads to protein and lipid peroxidation and damages the DNA strands, injuring the cells and leading to their death. The aim of the study was to evaluate the oxidative state in cats with hypertrophic cardiomyopathy and healthy controls.

**Results:**

In total, 30 cats divided into three groups were assessed: animals with clinically evident hypertrophic cardiomyopathy (HCM; *n* = 8), subclinical hypertrophic cardiomyopathy (SUB-HCM; *n* = 11) and healthy controls (*n* = 11). The activity of superoxide dismutase was statistically significantly lower in animals with symptomatic and asymptomatic hypertrophic cardiomyopathy (HCM 0.99 ± 0.35 U/mL; SUB-HCM 1.39 ± 0.4 U/mL) compared to healthy cats (2.07 ± 0.76 U/mL, *p* < 0.01). The activity of catalase was significantly lower in the SUB-HCM group (19.4 ± 4.2 nmol/min/mL) compared to the HCM (23.6 ± 5.9 nmol/min/mL) and the control (30 ± 7.5 nmol/min/mL, *p* < 0.01) group. The activity of glutathione peroxidase was 4196 ± 353 nmol/min/mL in the HCM group, 4331 ± 451 nmol/min/mL in the SUB-HCM group and 4037 ± 341 nmol/min/mL in the control group and did not differ significantly between groups. The total antioxidant capacity of plasma was 602 ± 65.5 copper reducing equivalents (CRE) in the HCM group, 605.9 ± 39.9 CRE in the SUB-HCM group and 629 ± 77.5 CRE in the healthy cats and did not differ significantly between the groups.

**Conclusions:**

Activities of superoxide dismutase and catalase differed in cats with hypertrophic cardiomyopathy, however the activity of the latter was only significantly lower in asymptomatic stage of the disease. The potentially beneficial effect of antioxidative substances on the disease progression in the asymptomatic and symptomatic stage of this disease should also be examined.

## Background

Hypertrophic cardiomyopathy (HCM) is the most common cardiovascular cause of death in cats [1]. The disorder involves symmetric or asymmetric left ventricular myocardial hypertrophy without any underlying risk factors. Although the majority of cats remain asymptomatic, some may develop signs of chronic heart failure due to diastolic failure, arterial thromboembolism (ATE) or sudden cardiac death [2, 3]. A particularly high incidence of hypertrophic cardiomyopathy in breeds such as the Maine Coon [4], Ragdoll [5], British shorthair [6] or Norwegian Forest cat [7] suggest a hereditary nature of the disease. To date, the genetic mutation of the cardiac myosin binding protein-C gene has only been identified in the Maine Coon and Ragdoll [5, 8]. Therefore, it is crucial to identify individuals that are in high risk of developing cardiac complications before the onset of life-threatening signs [9]. In the absence of consensus guidelines on the treatment of feline hypertrophic cardiomyopathy, all the therapeutic decisions are largely an extrapolation from human medicine. Currently, the therapy is aimed at prevention of congestive heart failure, arrhythmias and thromboembolic events [9, 10]. An inhibitor of sarcomere contractility, known as MYK-461, has been proven to inhibit the left ventricular hypertrophy, myocyte disarray and myocardial fibrosis in mice [11]. Thus far, only one report has been published on its efficacy in feline hypertrophic cardiomyopathy, showing its capability to reduce contractility, eliminate systolic anterior motion of the mitral valve and relieve left ventricular outflow tract obstruction [12].

Oxidative stress is the imbalance between the production and neutralisation of reactive oxygen species (ROS) [13]. Uncontrolled ROS overproduction leads to protein and lipid peroxidation and damages the DNA strands, injuring the cells and leading to their death. Living organisms have developed a number of mechanisms aimed at ROS detoxification. The main elements of the enzymatic system of antioxidant protection are superoxide dismutase (SOD) converting the superoxide anion (O2−) into hydrogen peroxide, which is ultimately detoxified by catalase (CAT) and glutathione peroxidase (GPx), with the water molecule as the end product [14]. Oxidative stress can also be determined based on the concentration of peroxidation byproducts. Malondialdehyde (MDA) is a well-known marker of serum lipid peroxidation. Oxygen free radicals have been proven to play a role in the pathogenesis of certain diseases. In such cases, the aim of the therapy is to supress oxidative stress and the damaging effects of free radicals. It has been proven that supplementation with antioxidative substances may have a positive effect on the course of those diseases, where excessive oxidative stress is associated with its pathogenicity [15]. One proven effect of antioxidants is the attenuation of the pacing-induced atrial remodelling or improvement in the endothelial function in patients with atherosclerosis [16, 17].

In the cardiovascular system, oxidative stress activates pathways responsible for pathological remodelling of the myocardium, impaired systolic function, fibroblast stimulation and the activation of metalloproteinases, which all contribute to chronic heart failure [18]. What is more, ROS can also stimulate cardiac hypertrophy via activation of kinase signalling pathways [19]. There is a paucity of information available in literature regarding oxidative stress in hypertrophic cardiomyopathy. One study showed increased serum levels of 8-isoprostaglandin F_2α_ in human patients with HCM compared to healthy controls and obstructive form of the disease was associated with higher levels of oxidative damage byproducts than non-obstructive subgroup [20]. Interestingly, an experimental study performed on guinea pigs showed, that heart muscle hypertrophy induced by ascending aorta banding is associated with decreased oxidative stress, however transition to heart failure stage was characterized by increased oxidative damage [21]. Only one study focused on feline HCM and an impaired cardiac mitochondrial oxidative phosphorylation capacity and increased mitochondrial ROS release were evidenced [22]. To date, blood parameters of oxidative stress and antioxidant defence mechanisms in the course of feline hypertrophic cardiomyopathy have not been studied.

The aim of the study was to evaluate the oxidative state in cats with hypertrophic cardiomyopathy and healthy controls.

## Results

In total, 30 cats were assessed. The mean (± standard deviation) age and weight of the animals was 4.6 ± 2.51 years and 5.36 ± 1.57 kg, respectively. The study population included 23 males, 17 of which were castrated, and seven females, 5 of which were spayed. Eleven healthy cats, with a mean age of 3.63 ± 2.06 years and a mean weight of 5.2 ± 1.54 kg, were included in the control group. That group included five Maine Coons, two European shorthair cats, two British shorthair cats, one ragdoll and one Norwegian forest cat. There were 11 cats in the asymptomatic hypertrophic cardiomyopathy group, nine of which were males. The mean age of the cats in this group was 5.94 ± 2.23 years, while the mean body weight was 4.95 ± 1.13 kg. Three British shorthair cats, two Maine Coons, two Devon Rexes, two European shorthair cats and one Sphinx, Persian and Scottish Fold cats each were included in that group. There were eight cats in the symptomatic hypertrophic cardiomyopathy group, seven of which were males. The mean age in that group was 6.1 ± 2.52 years, and the mean weight of the animals was 5.1 ± 2.04 kg. The group included three European Shorthair cats, two Maine Coons and one British Shorthair, Persian cat and Devon Rex each. There was no statistically significant difference between the age and the weight of the animals between the groups. The results of the complete blood count and serum biochemistry are presented in Table 1, and the cardiologic parameters are presented in Table 2.

**Table 1:** The results of the full blood count, blood biochemistry and hormone parameters of the studied animals divided into study groups

Variable	HCM	SUB-HCM	Control	*p* value
WBC [K/μL]	7.1 (5.17–13.99)	6.08 (4.08–18.94)	6.72 (4.28–10.69)	.67
RBC [M/μL]	9.04 (6.1–10.9)	9.31 (7.82–10.75)	9.42 (6.94–12.25)	.71
HGB [g/dL]	13.75 (8.7–15.2)	13.35 (11.8–15.5)	14.6 (12.1–17.3)	.44
HCT [%]	45.2 (26.5–51.8)	45.2 (39.7–49.4)	43.2 (36–64.1)	.82
AST [U/L]	40.5 (32–56)^Controls, SUB-HCM^	28 (15–47)^HCM^	22 (17–39)^HCM^	.001
ALT [U/L]	63.5 (44–143)^Controls^	58 (35–130)^Controls^	42 (17–39)^HCM, SUB-HCM^	.003
Urea [mmol/L]	9.95 (6.8–10.9)	7.9 (5–13.4)	7.7 (7–9.2)	.31
CREA [μmol/L]	156.5 (95–189)	141 (52–222)	156 (106–206)	.8
Mg [mmol/L]	0.95 (0.7–1.02)	0.81 (0.64–0.91)	0.81 (0.62–0.99)	.0573
Na^+^ [mmol/L]	152.3 (150.7–163.1)	151.7 (146.2–160.4)	153.2 (148.9–158.7)	.47
K [mmol/L]	4.17 (3.42–4.93)	3.93 (3.11–4.36)	4.17 (3.73–4.75)	.1
Cl^−^ [mmol/L]	116.5 (113.6–116.6)	117.5 (111.1–122.9)	118.5 (115.2–120.7)	.22
Ca^2+^ [mmol/L]	1.23 (1.19–1.33)	1.25 (1.1–1.76)	1.27 (1.09–1.61)	.51
glucose [mmol/L]	6.9 (4.5–11.9)	7.1 (5.7–15.1)	6.2 (4.6–13.8)	.55
total T4 [nmol/L]	22.08 (12.87–38.65)	26.93 (16.94–39.32)	30.73 (18.62–35.98)	.3

Data are presented as median (range)

**Table 2:** Echocardiographic parameters and the blood pressure of the studied animals divided into study groups

Variable	HCM	SUB-HCM	Control	*p* value
total number	8	11	11	*–*
LA/Ao	2.42 (1.42–4.53)^Controls^	1.6 (1.25–1.96)^Controls^	1.3 (0.88–1.48)^HCM, SUB-HCM^	.0001
LVIDd	14 (9–23.6)	12.8 (10.1–17.5)	13.9 (12.4–20.3)	.3
IVSd	6.9 (4.4–8.8)^Controls^	6.1 (3.9–7.5)^Controls^	4.2 (3–5.3)^HCM, SUB-HCM^	.0002
LVPWd	7.65 (4.9–8.3)^Controls^	5.8 (3.7–7.4)	4 (2.9–5.6)^HCM^	.0014
LVIDs	6.1 (2.2–12.8)	5.3 (3.6–9.1)	8.1 (5.4–11.3)	.07
IVSs	8.55 (5.8–10.2)	7.5 (5.7–9.3)	7 (5.3–9.3)	.36
LVPWs	9.95 (7.4–13.4)^Controls^	8.1 (6.4–10.5)	6.4 (5.1–9.6)^HCM^	.0012
LVOTO	4/8	4/11	0/11	.04
HR	203 (162–265)	222 (136–285)	214 (154–234)	.52
SAP [mmHg]	110 (95–136)^SUB-HCM^	139 (110–146)^HCM^	128 (90–150)	.03

Data are presented as median (range). Abbreviations: *LA/Ao* left atrium to aorta ratio, *LVIDd* left ventricular inner diameter in diastole, *IVSd* intraventricular septum in diastole, *LVOTO* left ventricular outflow tract obstruction, *HR* heart rate, *SAP* systolic arterial pressure. M-Mode heart dimensions were measured in the subvalvular region

The activity of superoxide dismutase (SOD) was statistically significantly lower in animals with symptomatic and asymptomatic hypertrophic cardiomyopathy (HCM 0.99 ± 0.35 U/mL; SUB-HCM 1.39 ± 0.4 U/mL) compared to healthy cats (2.07 ± 0.76 U/mL, *p* < 0.01). The activity of catalase (CAT) was significantly lower in animals at a pre-clinical stage of the disease (SUB-HCM; 19.4 ± 4.2 nmol/min/mL) and lower in symptomatic animals (HCM; 23.6 ± 5.9 nmol/min/mL) compared to healthy controls (30 ± 7.5 nmol/min/mL, *p* < 0.01), even though the latter difference was not statistically significant. The activity of glutathione peroxidase (GPx) was 4196 ± 353 nmol/min/mL in the HCM group, 4331 ± 451 nmol/min/mL in the SUB-HCM group and 4037 ± 341 nmol/min/mL in the control group and did not differ significantly between groups. The total antioxidant capacity of plasma was 602 ± 65.5 CRE in the HCM group, 605.9 ± 39.9 CRE in the SUB-HCM group and 629 ± 77.5 CRE in the healthy cats and did not differ significantly between the groups. In addition, there was no statistically significant difference between the groups in terms of the degree of lipid peroxidation – the concentration of malondialdehyde in blood serum was 4.07 ± 0.73 μM in the HCM, 3.84 ± 0.6 μM in the SUB-HCM group and 3.86 ± 0.71 μM MDA in the group of healthy animals.

The group of cats diagnosed with hypertrophic cardiomyopathy was subdivided into animals undergoing pharmacological treatment for the underlying medical condition (*n* = 9) and animals without cardiologic treatment (*n* = 10). The detailed characteristics of the pharmacological therapy are presented in Table 3. However, there were no statistically significant differences in the oxidative stress parameters in these subgroups.

**Table 3:** Type of pharmacotherapy received by the cats from the study groups

Therapy	no. of cats
asymptomatic hypertrophic cardiomyopathy (SUB-HCM)	5
atenolol	4
bisoprolol	1
symptomatic hypertrophic cardiomyopathy (HCM)	4
atenolol + furosemide	1
bisoprolol + furosemide	1
atenolol + furosemide + clopidogrel	2

Spearman correlation analysis found a significant moderate relationship between the activities of SOD and CAT (*r* = 0.44, *p* < 0.05).

## Discussion

This study assessed whether increased oxidative stress was a feature of symptomatic and asymptomatic hypertrophic cardiomyopathy. Oxidative stress has numerously been studied in felines and was discovered to be involved in pathogenesis of chronic renal failure [23], diabetes mellitus [24], feline infectious peritonitis [25] and immunodeficiency virus infection [26]. To the authors’ best knowledge, this is the first study that assesses blood parameters of oxidative stress in feline hypertrophic cardiomyopathy.

The activity of superoxide dismutase and catalase in the blood serum of the studied cats showed statistically significant differences between the groups. The serum activity of superoxide dismutase was significantly lower in the group of animals diagnosed with hypertrophic cardiomyopathy. This enzyme is an important component of the antioxidative enzyme system responsible for converting the superoxide radical anion (O2−) into H_2_O_2_, while one of its isoforms (SOD3) is present in blood serum [27]. This result is consistent with the findings of the study assessing a canine experimental model of heart failure caused by a surgically induced mitral valve insufficiency, where the activity of SOD in the left ventricular tissue was significantly lower in the studied animals than in the control group [28]. The results of studies on humans are divergent. The study assessing the extracellular SOD isofom in patients with cardiovascular disease of various origin [29] found that its activity was significantly lower compared to healthy controls, while this activity was significantly greater in patients with an acute coronary episode [30]. This may be explained by sudden periodic activation of the antioxidant enzyme system caused by acute ischemia. Interestingly however, cats with chronic renal failure had the same SOD acvitvity measured in erthrocyte lysate as healthy controls [23]. Hence, the activity of this enzyme may also be a sensitive indicator of oxidative stress in cats with cardiovascular disease. Catalase is one of the main antioxidative enzymes that catalyses the breakdown of H_2_O_2_, which is most active in erythrocytes and hepatocytes, although its antioxidant activity has also been described in blood serum [31, 32]. The activity of this enzyme was significantly lower in animals at a pre-clinical stage of the disease and lower in symptomatic animals, even though the latter difference was not statistically significant. In veterinary medicine, the activity of this enzyme in cardiovascular disease has not been studied. Human studies have found that the serum catalase activity gradually decreases with an increase in the number of diseases vessels [33].

This is partly compliant with the results of this study. The significant decrease in the catalase activity in the asymptomatic stage of the disease may be explained by the depletion of the anti-free radical protection mechanisms, while its increased activity in the symptomatic stage of cardiovascular disease may be caused by an activation of compensatory mechanisms in response to heart failure and excessive oxidative stress. Glutathione peroxidase is an antioxidative enzyme mainly involved in the detoxification of hydrogen peroxide, and one of its isoforms (GPx3) is present in blood serum [34]. In human medicine, its activity has been studied in the blood serum of cardiologic patients and was reported to be increased [35], decreased [36–38] or statistically unchanged [39, 40] compared to the other studied groups. Interestingly, in cats glutathione peroxidase concentrations were found to be significantly increased in the course of acute feline immunodeficiency virus infection [26]. This discrepancy may suggest disease-specific changes in the activity of this enzyme, as well as variations dependent on the methodology or the study design. The results of this study suggest that the extracellular GPx isofom may not be sensitive enough as a marker of oxidative stress in cats with hypertrophic cardiomyopathy, and examining its activity in other biological material, such as an erythrocyte lysate or heart tissue needs to be considered.

We did not observe statistically significant differences in the concentration of malondialdehyde (MDA), which is an indicator of lipid peroxidation, in blood serum of the studied animals. Numerous studies in humans found an increase in the concentration of MDA in patients with cardiovascular disease [41–43], and its association with the disease severity [42] and duration [43]. In contrast to those results, studies on dogs with dilated cardiomyopathy [44], and a mixed group of dogs with dilated cardiomyopathy and degenerative mitral valve disease [45, 46] found no statistically significant differences between groups, which is consistent with our findings. It has also been suggested that standard treatment of heart failure affects the degree of lipid peroxidation. One study on patients with heart failure undergoing pharmacological treatment found no increase in the concentration of MDA [47]. A lack of elevated MDA concentrations in animals suffering from cardiac disease may be caused by a different disease background (not caused by myocardial ischemia), species differences as well as ongoing pharmacological treatment. This prompts the study of 8-F_2α_-isoprostanes, which are the most valuable markers of lipid peroxidation [48].

Total antioxidant capacity (TAC) is a measure of free radical scavenging abilities of a substance. The assay used in our study utilized the cupric reducing antioxidant capacity (CUPRAC), which is chemically based on a single electron transfer mechanism. This assay does not measure antioxidant enzymes, but is focused on the measurement of thiol-group antioxidants, ascorbic acid, β- carotene, ɑ-tocopherol, uric acid, albumin, and bilirubin [49, 50]. The lack of differences between the studied groups may also be associated with the choice of a particular measurement method, although one study carried out on humans revealed a significant correlation between this method and other popular methods measuring TAC [51]. Uric acid is the main antioxidant in human serum, while it is present in much smaller quanitities in feline serum due to differences in purine metabolism in cats [52]. The studies carried out on dogs gave discrepant results: one study found a higher plasma antioxidant capacity in dogs with heart failure (oxygen radical absorbance capacity method) [46], while the second study using the ferric reducing antioxidant power method confirmed the findings of the first study, although the total antioxidant capacity did not differ between the studied groups of dogs using the Trolox equivalent antioxidant capacity method [53]. Therefore, despite the presence of a correlation between the different methods used to measure TAC, it seems that the choice of a suitable method is essential.

It has been found that numerous drugs used to treat hypertrophic cardiomyopathy, such as ACE inhibitors, beta-blockers and calcium channel blockers, may have antioxidant effects [54–56]. Contrary to our expectations, we did not find statistically significant differences in any of the oxidative stress parameters between cats without treatment and those in the course of pharmacological therapy. This result is consistent with the findings in dogs with cardiovascular disease [45]. The groups in the current study were small, and the pharmacotherapy was tailored to the patient needs and differed between individuals. In addition, the current study was not specifically designed to assess this matter, which may have affected the results.

Our study had some limitations. It is clear that small sample size is a major limitation of our study. In future studies, this number should be higher in order to extrapolate to a larger population. The impact of the diet of the studied animals on the oxidative stress parameters cannot be ruled out. However, all the cats were fed commercial feline diets. The body score was not assessed when including the cats in the study although evidently obese or thin individuals were excluded from it. No uniform pharmacological therapy of the animals was another major study limitation.

## Conclusions

In conclusion, the results indicate that the activities of superoxide dismutase and catalase are different in cats with hypertrophic cardiomyopathy, however the activity of the latter was only lower in asymptomatic stage of the disease. However, further studies assessing other markers of oxidative stress and using other biological tissues of cats with hypertrophic cardiomyopathy are warranted. In addition, the potentially beneficial effect of antioxidative substances on the disease progression in the asymptomatic and symptomatic stage of this disease should also be examined.

## Methods

### Animals

This observational, cross-sectional study was performed with 30 cats that were divided into two groups: hypertrophic cardiomyopathy and control group. Before enrolling an animal into the study, a written consent for a full cardiac examination and blood sampling was obtained from the owner. The cats remained under the care of their owners during and after the study.

All cats included in the study underwent a clinical examination, a six-lead electrocardiogram (BTL-08, BTL Industries, U.K.), a standard thransthoracic echocardiography (Aloka F36 or Aloka Arietta V60, Hitachi-Aloka, Tokyo, Japan), and a non-invasive blood pressure measurement using the Doppler ultrasonic method (Parks 811-B, Eickemeyer Veterinary Equipment Inc., Germany). A full blood count (LaserCyte Dx, IDEXX Laboratories, Westbrook, ME, USA) and blood chemistry (Konelab Prime 30ISE, Thermo Scientific, Waltham, USA) including the T4 concentration (miniVidas, bioMérieux, Marcy l’Etoile, France) were carried out. The full cardiac examination was performed without sedation in a quiet room.

The hypertrophic cardiomyopathy group consisted of 19 animals diagnosed with hypertrophic cardiomyopathy by means of transthoracic echocardiography. The inclusion criteria for this group were as follows: an increase in the interventricular septal wall thickness at end-diastole (IVSd) and/or an increase in the left ventricular free wall thickness at end-diastole (LVPWd) exceeding the cut-off value of 6 mm, as previously described [1]. In all cats diagnosed with HCM, a thoracic radiography (GIERTH HF 200A; Gierth X-Ray International GmbH, Riesa, Germany) was performed.

On the basis of clinical manifestations and radiographic findings the affected cats were divided into two groups: asymptomatic hypertrophic cardiomyopathy (SUB-HCM) and symptomatic hypertrophic cardiomyopathy (HCM). Cats included in HCM group presented signs of congestive heart failure, which was diagnosed based on radiographic evidence of pulmonary edema and/or pleural effusion and clinical signs of dyspnea. Some of the animals with hypertrophic cardiomyopathy were already treated before the blood was collected for testing with standard protocol for the disease, which included loop diuretics, beta-blockers and antiplatelet drugs, as indicated in Table 3 [10]. The therapy was not uniform and individually tailored for the needs of each animal.

The control cats consisted of 11 clinically healthy cats of various breeds presenting to Faculty’s Small Animal Veterinary Practice for preventive screening. Additional cardiac examination was offered to these individuals at no further cost. These cats were deemed healthy of the basis of history, clinical examination, laboratory, echocardiographic and blood pressure measurement findings.

Animals presenting clinical symptoms of any other disease, clearly skinny or obese, suspected of hyperthyroidism (serum thyroxine concentration > 50 nmol/L), hypertension (systolic blood pressure > 160 mmHg) or diabetes mellitus (plasma glucose concentration ≥ 280 mg/dL) were excluded from the study. In symptomatic animals, a twofold increase in the reference values of aminotransferases and blood urea above the reference values were considered acceptable and occurred as a result of congestive heart failure and prerenal azotemia.

### Echocardiography

Transthoracic echocardiography was performed by experienced echocardiographers (UP and ANN) using an echocardiograph equipped with a 3–14 MHz sector, phased-array transducer. The LA/Ao ratio and M-mode echocardiography measurements were performed using the right parasternal short-axis view. Hypertrophic cardiomyopathy was diagnosed when M-Mode derived IVSd and/or LVPWd measuremens were ≥ 6 mm [1]. Diagnosis of hypertrophic obstructive cardiomyopathy was made by the presence of left ventricular hypertrophy together with one of the following signs: systolic anterior motion of the mitral valve leaflet noted on M-mode echocardiography, mitral valve regurgitant flow and dynamic left ventricular outflow tract obstruction evident on color flow Doppler echocardiography and/or an increased peak with a dagger-shaped spectrum in the left ventricular outflow tract velocity apparent on continuous wave Doppler echocardiography [57].

### Blood pressure measurement

Systolic arterial blood pressure measurement was taken using a noninvasive Doppler method and a standardized ISFM protocol was adhered to [58]. Measurements were taken in a calm and quiet environment with only owner and experienced examiner present and away from other animals. All cats were allowed to acclimatise for a minimum of 5 min. An appropriately sized inflatable cuff was placed around forelimb and at least 6 measurements were taken. Systolic blood pressure was calculated as mean of several consistent readings.

### Blood analyses

Blood samples were collected from the cephalic vein into plain and lithium heparin tubes as a part of the cardiac examination. Blood collected into plain tubes was left to clot for 15 min in room temperature and was then centrifuged at 2000 x g at 4 °C for 15 min, while blood collected into lithium heparin tubes was centrifuged immediately after collection in the same conditions. To avoid the influence of leukocytes or red blood cells antioxidant enzymes activity, plasma and serum were collected from top layer without disturbing a buffy coat layer. Plasma and serum was transferred to an analytical laboratory for basic laboratory tests, while the remaining material was divided and immediately frozen at − 80 °C until analysis. All enzymatic tests were assayed within 4 weeks of sample collection. Laboratory tests included haematology and biochemical analysis: alanine transaminase (ALT), aspartate transaminase (AST), urea, creatinine, glucose, total thyroxine (T4) and ionogram (Mg^2+^, Cl^−^, K^+^, Na^+^, Ca^2+^).

#### Determination of sera antioxidant enzymes activity

##### Superoxide dismutase

The serum activity of CuZn-SOD was determined using a commercially available assay kit (Cayman Chemical, Ann Arbor, MI; 706002). To validate it for feline serum, the within-run precision was estimated by calculating the intra-assay coefficient of variation (CV) on the basis of the results obtained after performing the tests in 3 samples repeated 2 times. Mean coefficient of variation for 1:5 dilution was 24%. The linearity for test was assessed using three serum samples (one for each group). Samples were diluted 1:1, 1:5, 1:25 and 1:125 in samples buffer and tested. A correlation analysis was used to examine the relationship results of diluted samples and expected results and the coefficient of regression (R2) was calculated. Dilution of 1:5 was closest to the fitted regression line. The serum was diluted and analyzed according to the manufacturer’s instruction. One unit was defined as the amount of enzyme needed to elicit 50% dismutation of the superoxide anion radical.

##### Catalase

The activity of serum catalase was measured using a commercially available kit (Cayman Chemical; 707002). Because erythrocytes are rich in CAT, serum samples have been prepared with high precision and have not been used if any signs of haemolysis was observed. Assay serum dilution was tested and validated in the same way as for *superoxide dismutase*. Intra-assay coefficient of variation of undiluted serum was 20%. We used undiluted serum to run the assay in accordance with the manufacturer’s instruction. Feline serum exhibited a linear relationship between the amount of sample and CAT activity over a wide range. One unit was defined as the amount of enzyme causing the formation of 1 nmol of formaldehyde per minute at room temperature.

##### Glutathione peroxidase

The plasma glutathione peroxidase enzyme assay was carried out according to the Paglia and Valentine method using Cayman’s assay kit (Cayman Chemical; 703102). The plasma dilution was validated and intra-coefficient of variation was calculated (7.65%). Samples were diluted 1:2 in sample buffer provided by producent. Activity of enzyme was calculated as a change in absorbance during one minute. GPx activity was calculated using a formula:

$$ GPX\  activity=\frac{\Delta  A340/\mathit{\min}}{0.00373{\mu M}^{-1}}\bullet \frac{0,19\  ml}{0,02 ml}\bullet 2=\frac{nmol}{\mathit{\min}}/ ml $$

One unit of activity was defined as the amount of enzyme causing the oxidation of 1 nmol of NADPH to NADP+ per minute per mL of sample at room temperature.

#### Determination of plasma total antioxidant capacity

The total antioxidant capacity in the heparinized plasma was determined using the OxiSelect Total Antioxidant Capacity assay kit (Cell Biolabs Inc., San Diego, CA, USA). Test was validated for feline plasma and intra-coefficeient of variation was calculated (5.7%). The result was calculated and the total sample antioxidant power was expressed as copper reducing equivalents (CRE).

#### Determination of total malondialdehyde in plasma (MDA)

Lipid peroxidation levels in the heparinized plasma were assessed by measuring malondialdehyde (MDA) formation using the Bioxytech MDA-586 kit (Oxis Research, Portland, OR, USA). Precision of the test for feline plasma was 16.7%. Undiluted samples were prepared following the instructions of the manufacturer. To prevent further sample oxidation, the hydrolysis of the samples was performed in the presence of butylated hydroxytoluene). The results are shown as the concentration of MDA in the sample, expressed in μM.

The absorbance of the products of all the enzymatic reactions were measured spectrophotometrically using a microplate reader (Tecan Spark 10 M, Tecan, Austria). All the samples were assayed in the same assay run in duplicate and analysed simultaneously by one examiner (AT), who was blinded to the sample origin, while the final result was an average value of two measurements.

#### Statistical methods

All the collected data was subjected to statistical analysis using GraphPad Prism 5.0 software. The D’Agostino and Pearson normality test were used to assess the distribution of the data. Depending on the results, the groups were compared using the one-way ANOVA with Bonferroni post-hoc test or the Kruskal-Wallis with the post-hoc Dunns analysis. An unpaired t-test or the Mann-Whitney test were used to compare two sets of variables, depending on the data distribution. The correlation between the groups of variables was determined using the Spearman correlation coefficient. Statistical significance was set at *p* ≤ 0.05.

## Data Availability

The datasets used and/or analysed during the current study are available from the corresponding author on reasonable request.

## Abbreviations

ALT: Alanine transaminase
AST: Aspartate transaminase
ATE: Arterial thromboembolism
CAT: Catalase
CRE: Copper reducing equivalents
GPx: Glutathione peroxidase
HCM: Hypertrophic cardiomyopathy
MDA: Malondialdehyde
ROS: Reactive oxygen species
SOD: Superoxide dismutase
T4: Thyroxine
TAC: Total antioxidant capacity
